# Genetic architecture of cardiometabolic risks in people living with HIV

**DOI:** 10.1186/s12916-020-01762-z

**Published:** 2020-10-28

**Authors:** Haoxiang Chang, Anshuman Sewda, Carla Marquez-Luna, Sierra R. White, Bridget M. Whitney, Jessica Williams-Nguyen, Robin M. Nance, Won Jun Lee, Mari M. Kitahata, Michael S. Saag, Amanda Willig, Joseph J. Eron, W. Christopher Mathews, Peter W. Hunt, Richard D. Moore, Allison Webel, Kenneth H. Mayer, Joseph A. Delaney, Paul K. Crane, Heidi M. Crane, Ke Hao, Inga Peter

**Affiliations:** 1 Department of Genetics and Genomic Sciences, Icahn School of Medicine at Mount Sinai, 1425 Madison Avenue, New York, NY 10029 United States of America; 2 Institute of Health Management Research, IIHMR University, Jaipur, Rajasthan India; 3 The Charles Bronfman Institute for Personalized Medicine, Icahn School of Medicine at Mount Sinai, New York, NY United States of America; 4 Department of Epidemiology, University of Washington School of Public Health, Seattle, WA United States of America; 5 Department of Medicine, University of Washington School of Medicine, Seattle, WA United States of America; 6 Center for AIDS Research, University of Washington, Seattle, WA United States of America; 7 School of Medicine, University of Alabama at Birmingham, Birmingham, AL United States of America; 8 Department of Medicine, School of Medicine, University of North Carolina at Chapel Hill, Chapel Hill, NC 27514 United States of America; 9 Department of Medicine, University of California San Diego, San Diego, CA United States of America; 10 Division of Experimental Medicine, University of California San Francisco, San Francisco, CA United States of America; 11 Department of Medicine, Johns Hopkins University, Baltimore, MD United States of America; 12 Department of Epidemiology, |Johns Hopkins University, Baltimore, MD United States of America; 13 Frances Payne Bolton School of Nursing, Case Western Reserve University, Cleveland, OH United States of America; 14 The Fenway Institute at Fenway Health, Boston, MA United States of America

**Keywords:** HIV, Polygenic risk score, Lipoprotein, Triglyceride, Type 2 diabetes, Myocardial infarction, Genome-wide association study

## Abstract

**Background:**

Advances in antiretroviral therapies have greatly improved the survival of people living with human immunodeficiency virus (HIV) infection (PLWH); yet, PLWH have a higher risk of cardiovascular disease than those without HIV. While numerous genetic loci have been linked to cardiometabolic risk in the general population, genetic predictors of the excessive risk in PLWH are largely unknown.

**Methods:**

We screened for common and HIV-specific genetic variants associated with variation in lipid levels in 6284 PLWH (3095 European Americans [EA] and 3189 African Americans [AA]), from the Centers for AIDS Research Network of Integrated Clinical Systems cohort. Genetic hits found exclusively in the PLWH cohort were tested for association with other traits. We then assessed the predictive value of a series of polygenic risk scores (PRS) recapitulating the genetic burden for lipid levels, type 2 diabetes (T2D), and myocardial infarction (MI) in EA and AA PLWH.

**Results:**

We confirmed the impact of previously reported lipid-related susceptibility loci in PLWH. Furthermore, we identified PLWH-specific variants in genes involved in immune cell regulation and previously linked to HIV control, body composition, smoking, and alcohol consumption. Moreover, PLWH at the top of European-based PRS for T2D distribution demonstrated a > 2-fold increased risk of T2D compared to the remaining 95% in EA PLWH but to a much lesser degree in AA. Importantly, while PRS for MI was not predictive of MI risk in AA PLWH, multiethnic PRS significantly improved risk stratification for T2D and MI.

**Conclusions:**

Our findings suggest that genetic loci involved in the regulation of the immune system and predisposition to risky behaviors contribute to dyslipidemia in the presence of HIV infection. Moreover, we demonstrate the utility of the European-based and multiethnic PRS for stratification of PLWH at a high risk of cardiometabolic diseases who may benefit from preventive therapies.

## Background

The number of people living with human immunodeficiency virus (HIV) infection (PLWH) worldwide has increased by 34.6% (from 27.4 million to 36.9 million) between 2000 and 2018, while acquired immune deficiency syndrome (AIDS)-related deaths have declined from 1.5 million to 940,000 annually [1]. These advances can be primarily attributed to therapeutic advances in antiretroviral therapy (ART) and improved access to ART, allowing PLWH to live longer. However, accumulating evidence suggests that PLWH are at a higher risk of cardiovascular diseases (CVD) and have increased CVD-related mortality rates than those without HIV [2–6]. The possible causes of increased CVD risk among PLWH include inflammation and immune activation in response to HIV infection and viremia, adverse effects of ART, and lifestyle risk factors (e.g., smoking, alcohol, and illicit drug use). However, these factors do not fully account for the increased risk of CVD in PLWH [7, 8].

Genetic variants have been identified as significant predictors of traditional CVD risk factors including cardiometabolic traits and diseases, such as dyslipidemia and lipid levels (low-density lipoprotein cholesterol (LDL), high-density lipoprotein cholesterol (HDL), and triglycerides) [9, 10], obesity [11, 12], type 2 diabetes mellitus (T2D) [13], and myocardial infarction (MI) [14] in the general population. CVD and related disorders have been demonstrated to have polygenic modes of inheritance, meaning that common genetic variants with small effect sizes located in multiple genes contribute to variability in disease or trait risk [15, 16]. Polygenic risk scores (PRS) have been proposed to assess the cumulative burden of multiple common susceptibility loci [17, 18]. A recent study found that 8% of the population possesses a genetic predisposition that confers a more than three-fold increased risk for coronary artery disease (CAD), with the highest PRS percentiles identifying 20 times more people than found by familial hypercholesterolemia mutations at a comparable or higher risk [19–21]. Moreover, in randomized clinical trials, people with the highest burden of genetic risk demonstrated the most substantial clinical benefit from primary prevention (statin therapy) resulting in a roughly three-fold decrease in the number needed to treat to prevent one CAD event [22].

Despite the growing literature proposing the clinical value of PRS in the general population [23], only a few reports with limited sample sizes have demonstrated the contribution of genetic variation to cardiometabolic risk in PLWH [24–26]; even fewer have examined the utility of PRS in PLWH [27]. Therefore, this study aimed to identify genetic predictors of cardiometabolic traits in PLWH and systematically assess the performance of PRS derived using results from previously published well-powered genome-wide association studies (GWAS) of T2D [28], CAD [29, 30], lipids (LDL, HDL, and triglyceride levels) [31], and body mass index (BMI) [32], and genomic data from the largest ethnically diverse PLWH cohort to date with genetic information. Given the emerging interest in applying PRS to improve clinical decision making [33], this study may help shed light on the genetic predictors of cardiometabolic risk in the presence of HIV infection and improve risk stratification to identify individuals at a high risk of CVD.

## Methods

### Study participants

The Centers for AIDS Research Network of Integrated Clinical Systems (CNICS) cohort includes a multiethnic population of ~ 36,000 PLWH (age 18 years and older) who have received routine clinical care at one of eight sites in the USA [34]. CNICS has an ongoing genetics project in which adult PLWH across racial/ethnic backgrounds from all sites, who provided informed consent and contributed specimens to the CNICS biospecimen repository, are being genotyped. Study participants were included if their genetic data were available at the time of these analyses.

### Measurement of cardiometabolic phenotypes

The CNICS data repository integrates comprehensive clinical data from sites from outpatient and inpatient encounters, including information on demographic characteristics, clinical and laboratory data, medications, and historical clinical information. Lipid levels in CNICS include HDL, LDL, and triglyceride values measured as part of routine care and, therefore, may or may not have been obtained in the fasting state. LDL was either measured directly or calculated using the Friedewald equation [35]. BMI was calculated from heights and weights as a continuous variable (kg/m^2^). PLWH were categorized as ART-naïve or experienced. Among participants, the initial CNICS visit dates ranged from 1995 to 2015. Between the initial and the last CNICS visits, the average follow-up period was 10.3 years (median, 9.9 years; range, 0–23 years). Most included PLWH had multiple recorded values for each lipid drawn as part of care, we used mean values. We excluded individuals who were taking lipid-lowering drugs (e.g., HMG Co-A reductase inhibitors or statins) at baseline.

T2D diagnosis in CNICS is based on the following criteria: (1) hemoglobin A1c ≥ 6.5; (2) use of a diabetes-specific medication such as insulin; or (3) use of a diabetes-related medication, which is frequently, but not exclusively, used to treat diabetes (e.g., biguanides) in the setting of also having a diabetes diagnosis [36]. We have found high sensitivity (99%) and specificity (97%) for this definition [36].

CNICS uses an established state-of-the-art approach to adjudicate [37, 38] and classify MIs based on the Universal Definition of MIs [39, 40]. Potential MIs in the centralized CNICS data repository were identified using a comprehensive set of MI diagnostic and procedure codes and elevated cardiac biomarker values to optimize the ascertainment sensitivity as previously described [37, 38]. De-identified packets were prepared that contained provider notes, electrocardiograms, laboratory reports, and results from imaging and procedures, such as cardiac catheterization. Two physicians with expertise in adjudicating cardiac events performed a centralized review of the patient data, followed by inputs from a third physician for resolving discrepancies. We included type 1 MIs, those due to atheroembolic disease, and excluded type 2 MIs due to a mismatch in the oxygen supply and demand, usually observed in the setting of sepsis or cocaine or other illicit drug-induced vasospasm [37].

### Genotyping and imputation

DNA was isolated from peripheral blood mononuclear cells or buffy coats of PLWH obtained from the CNICS biorepository using the FlexiGene DNA kit (Qiagen, #51206). DNA samples were then normalized and genotyped using Illumina’s high-density custom Multiethnic Global Array (MEGA) series BeadChips. Genotyped variant calling was performed using GenomeStudio® Genotyping Module v2.0 software (Illumina®, San Diego, California, USA) and zCall [41]. PLINK v.1.9 was used to exclude single nucleotide polymorphisms (SNPs) with call rates < 95%, minor allele frequency < 1%, and deviation from Hardy-Weinberg equilibrium (*p* value <1E-5), as well as samples with call rates < 90%, sex discrepancies between genotype data and self-report, and pairwise identity-by-descent (pi-hat > 0.9) [42].

We inferred ethnicity on genotype data using GRAF-pop software [43], and, after excluding the human leukocyte antigen encoding region, performed principal components analysis (PCA) on the African American (AA) and European American (EA) samples separately using EIGENSOFT [44]. The estimated principal components (PCs) were included in the regression models while performing genome-wide association analysis in each ancestry group. Genotype data from each ancestry group was imputed separately using the cloud-based Michigan Imputation Server [45] and Trans-Omics for Precision Medicine, or TOPMed data, as the reference panel (https://www.nhlbiwgs.org/). For further analysis, we only kept variants that were imputed with high quality (imputation quality score, *r*^2^ > 0.3) and passed the standard quality control procedures. The genotyped and imputed SNP counts are listed in Additional file 1: Table S1.

### Genome-wide association analysis

Genome-wide association tests were conducted on each SNP using either linear or logistic regression method on imputed dosage data sets, using in-house code written in *R* (version 3.5.3). The tests were performed separately in European and African ancestry sub-cohorts, and then pooled using random-effects meta-analysis, implemented in the “meta” *R* package [46]. In addition to the first ten PCs, analyses were adjusted for site, age, sex at birth, and presence or absence of ART. A study reported that genetic associations with lipid traits differed by sex [47]; therefore, we repeated these analyses in male and female sub-cohorts separately. The results were visualized through multi-phenotype and single-phenotype mirrored Manhattan plots. HIV-specific genetic variants were defined as loci that were significant at *p* < 0.01 in GWAS_HIV_ and had *p* ≥ 0.05 in the well-powered GWAS_GEN_, and the 99% confidence intervals (CI) for the beta coefficients in GWAS_HIV_ and GWAS_GEN_ did not overlap. Similar approach was used to detect ancestry-specific or sex-specific lipid-related variants.

### Gene set enrichment analysis

Enrichr was used to perform gene set enrichment analyses using the genes containing HIV-specific variants. Enrichr database is an integrative web-based application, currently containing 335,434 annotated gene sets from 166 gene set libraries [48, 49]. UK Biobank consists of a large prospective cohort of more than 500,000 middle-aged participants with detailed information on a wide range of complex diseases, lifestyle risk factors, medical history, and physical measurements [50]. The health outcomes were adjudicated by experts for a range of disease areas. The genetic data and statistical analyses were synchronized across multiple phenotypes. We looked for enrichment in the UK Biobank GWAS version 1 (https://www.ukbiobank.ac.uk/tag/gwas/) gene set library which contains 857 terms covering 14,148 genes (122 genes per term). Adjusted *p* values calculated using the false discovery rate (FDR) for correction for multiple hypotheses testing [51] were reported for each term. An adjusted *p* < 0.05 was considered statistically significant.

### Expression quantitative trait loci (eQTL) analysis

To assess the functional relevance of the newly observed associations, we tested whether HIV-specific loci are enriched among variants shown to regulate gene expression (eQTLs). We acquired eQTL data in primary CD14+ human monocytes from 432 European volunteers at baseline and after exposure to the inflammatory proxies interferon-γ (IFN-γ) or differing durations (2 h or 24 h) of lipopolysaccharide (LPS), which was profiled using the Illumina Human OmniExpress BeadChips genotyping array [52]. SNPs that were significantly associated with each trait at *p* < E−6 in GWAS_GEN_ of lipid profiles were excluded [31]. Furthermore, linkage disequilibrium (LD)-based pruning was performed using a threshold of *r*^2^ > 0.2. After variant-filtering, we used chi-squared tests to compare the proportion of the eQTL SNPs (eSNPs) that were associated with gene expression levels at 10% FDR, among the HIV-specific loci to the remaining non-significant SNPs.

### Polygenic risk score analysis

#### Traditional PRS

The PRS, representing estimated genetic determinants for five traits (HDL, LDL, triglycerides, T2D, and type 1 MI) were computed following the thresholding-pruning procedure [53]. We computed PRS for EA sub-cohort of PLWH (PLWH_EA_) and AA sub-cohort of PLWH (PLWH_AA_) separately using linear combinations of the imputed genotype dosages [54], and regression coefficients from the respective summary association statistics retrieved from previously published GWAS conducted in the general population largely of European ancestry: Global Lipids Genetics Consortium (GLGC) [31]; Genetic Investigation of ANthropometric Traits (GIANT) consortium [32]; DIAbetes Genetics Replication And Meta-analysis (DIAGRAM) consortium [28]; Coronary ARtery DIsease Genome wide Replication and Meta-analysis plus the Coronary Artery Disease Genetics (CARDIoGRAMplusC4D) consortium [29]; and UKBiobank CardioMetabolic Consortium [30] (PRS_GEN,_ Additional file 1: Table S2). For each disease/trait, we calculated eight sets of PRS using GWAS *p* value thresholds of 1E−1, 1E−2, 1E−3, 1E−4, 1E−5, 1E−6, 1E−7, and 1E−8 for including SNPs in the PRS derivation. Prior to the calculation for each threshold, the retrieved SNPs underwent LD-based pruning using the 1000 Genomes European and African reference populations [55] as implemented in PLINK, and highly redundant SNPs (*r*^2^ ≥ 0.5) were removed (see Additional file 1: Table S3 for the number of SNPs used to calculate each PRS). For each *p* value threshold, we tested associations between PRS from previously reported GWAS (Additional file 1: Table S2) and the trait of interest or disease case status and visualized it using a heatmap.

#### Multiethnic PRS

To derive PRS that would perform well for both PLWH_EA_ and PLWH_AA_, we considered GWAS summary statistics from two training sources: (1) the GWAS conducted in the general population of European ancestry (PRS_EA_) and (2) the GWAS conducted in PLWH_AA_ (PRS_AA_), using ten-fold cross-validation. Additionally, we derived multiethnic PRS (Additional file 1**:** Table S3) that combined the two training sources using a recently published method [56]. Briefly, the multiethnic PRS is defined as the linear combination of the two PRSs with mixing weights *α*_1_ and *α*_2_. That is,

$$ {\mathrm{PRS}}_{\mathrm{EA}+\mathrm{AA}}={\alpha}_1{\mathrm{PRS}}_{\mathrm{EA}}+{\alpha}_2{\mathrm{PRS}}_{\mathrm{AA}} $$

We estimated mixing weights *α*_1_ and *α*_2_ using validation data by fitting a linear regression model and computed adjusted *R*^2^ to account for the additional degree of freedom. We employed a ten-fold cross-validation, using 90% of the cohort to estimate GWAS regression coefficients and the remaining 10% of the cohort to validate predictions (using the adjusted-*R*^2^ metric with best-fit mixture weights, ${\hat{\alpha}}_1$ and ${\hat{\alpha}}_2$) and reported an average adjusted *R*^2^ across the ten cross-validations. For each fold, we computed regression coefficients using linear regression for quantitative traits while adjusting for 10 PCs, sex, age, age^2^, presence or absence of ART, and site, where the PCs were estimated using only PLWH_AA_. For T2D and MI diagnoses that had low prevalence in our cohort, we used stratified ten-fold cross-validation, where each cross-validation had the same case-control ratio. For lipid traits, for each *p* value threshold, we calculated the *R*^2^ statistic derived from a fixed-effects meta-analysis of marginal associations between PRS_EA + AA_ and the trait of interest.

Lastly, we estimated the prevalence of T2D and MI for PLWH with the highest European-based and multiethnic PRS. We applied multiple testing correction to account for the number of thresholds and PRS tested using FDR [51]. An adjusted *p* < 0.05 was considered statistically significant. The number of SNPs used to calculate various multiethnic PRS is reported in Additional file 1: Table S3.

## Results

The final cohort consisted of 6284 PLWH with 3095 PLWH_EA_ and 3189 PLWH_AA_; both sub-cohorts were predominantly male (89% and 69%, respectively), which is consistent with the HIV epidemic in the USA (Table 1). PLWH_AA_ had a higher prevalence of T2D (*p* < 0.0001, Table 1), but lower mean LDL (*p* < 0.0001) and triglyceride (*p* < 0.0001) levels and higher mean HDL levels (*p* < 0.0001) than PLWH_EA_ (Table 2).

**Table 1:** Baseline demographic and clinical characteristics of the study cohort

Variable	PLWH_EA_^a^ *n* = 3095 (%)	PLWH_AA_^b^ *n* = 3189 (%)	Total *N* = 6284 (%)	*p* value^c^
Age	53.36 ± 9.70	53.18 ± 10.70	53.27 ± 10.22	0.49
Gender				< 0.0001
Male	2763 (89.3)	2199 (69.0)	4962 (79.0)	
Female	332 (10.7)	990 (31.0)	1322 (21.0)	
Site				< 0.0001
University of Alabama	744 (24.0)	882 (27.7)	1626 (25.9)	
Johns Hopkins	135 (4.4)	845 (26.5)	980 (15.6)	
University of Washington	623 (20.1)	261 (8.2)	884 (14.1)	
University of California San Diego	640 (20.7)	187 (5.9)	827 (13.2)	
Case Western Reserve University	314 (10.1)	494 (15.5)	808 (12.9)	
University of North Carolina	161 (5.2)	368 (11.5)	529 (8.4)	
Fenway	309 (10.0)	45 (1.4)	354 (5.6)	
University of California San Francisco	169 (5.5)	107 (3.4)	276 (4.4)	
Type 2 diabetes^d^	388 (12.5)	676 (21.2)	1064 (16.9)	< 0.0001
Myocardial infarction^e^	53 (1.7)	64 (2.0)	117 (1.9)	< 0.39
CD4 counts^c^	399 ± 283.5	331 ± 277.3	364 ± 282.4	< 0.0001
Presence of antiretroviral therapy	2841 (91.8)	2823 (88.5)	5664 (90.1)	< 0.0001

^a^PLWH_EA_, European American sub-cohort of people living with HIV. ^b^PLWH_AA_, African American sub-cohort of people living with HIV. ^c^The *p* values were calculated using a *t*-test. ^d^During study follow-up. ^e^At baseline

**Table 2:** Mean (standard deviation) and mean comparison *p* values for lipid values stratified by European American vs. African American race in the study cohort

		PLWH_EA_^a^		PLWH_AA_^b^		
Trait	Subgroup	*n*	Mean (standard deviation)	*n*	Mean (standard deviation)	*p* value^c^
HDL	Pooled	3095	41.35 (13.22)	3189	48.36 (15.44)	< 0.0001
	Female	332	47.51 (15.47)	990	52.98 (16.57)	< 0.0001
	Male	2763	40.61 (12.72)	2199	46.29 (14.44)	< 0.0001
	*p* value^d^	.	< 0.0001	.	< 0.0001	.
LDL	Pooled	2926	107.6 (31.20)	3138	100.1 (32.99)	< 0.0001
	Female	317	107.2 (29.46)	975	103.6 (33.93)	0.0689
	Male	2609	107.6 (31.41)	2163	98.58 (32.45)	< 0.0001
	*p* value^d^	.	0.839	.	< 0.0001	.
Triglycerides	Pooled	3083	206.7 (171.7)	3175	155.6 (103.3)	< 0.0001
	Female	331	185.2 (141.1)	986	142.3 (76.86)	< 0.0001
	Male	2752	209.3 (174.8)	2189	161.60 (112.8)	< 0.0001
	*p* value^d^	.	0.0045	.	< 0.0001	.

^a^PLWH_EA_, European American sub-cohort of people living with HIV. ^b^PLWH_AA_, African American sub-cohort of people living with HIV. HDL, high-density lipoproteins, LDL, low-density lipoproteins. ^c^*p* values for differences in each continuous variable by race. ^d^*p* values for differences in each continuous variable by gender

**Fig. 1:**
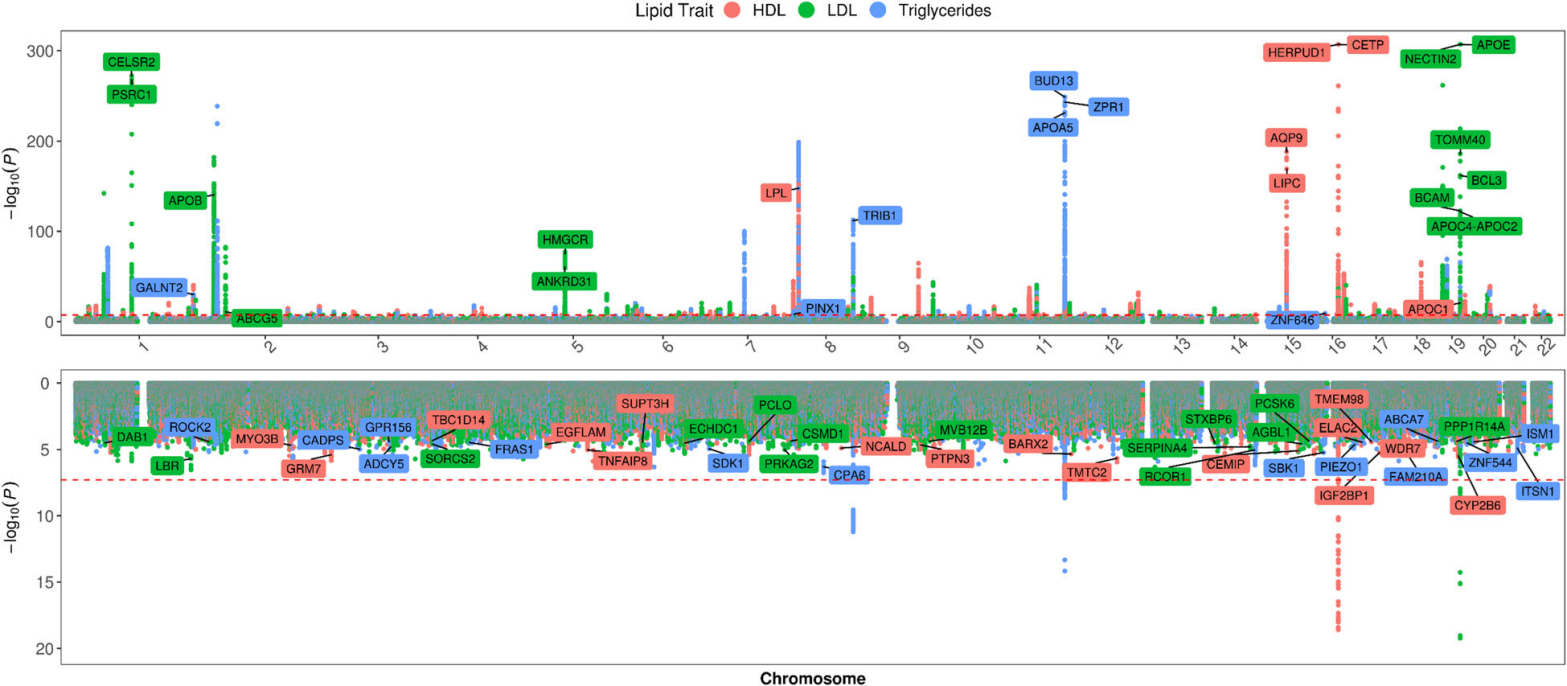
Multi-phenotype, mirrored Manhattan plot of genome-wide association analysis of lipid traits in Willer et al. [31] (top) and the CNICS European American (bottom) cohorts. HDL, high-density lipoproteins, LDL, low-density lipoproteins. In the top panel, gene names are listed for loci with association *p* < E−5 in both cohorts. In the bottom panel, gene names are listed for loci if *p* < 0.01 in the CNICS cohort and *p* > 0.05 in the Willer et al. cohort and there is no overlap between 99% confidence intervals for the corresponding beta coefficients

**Fig. 2:**
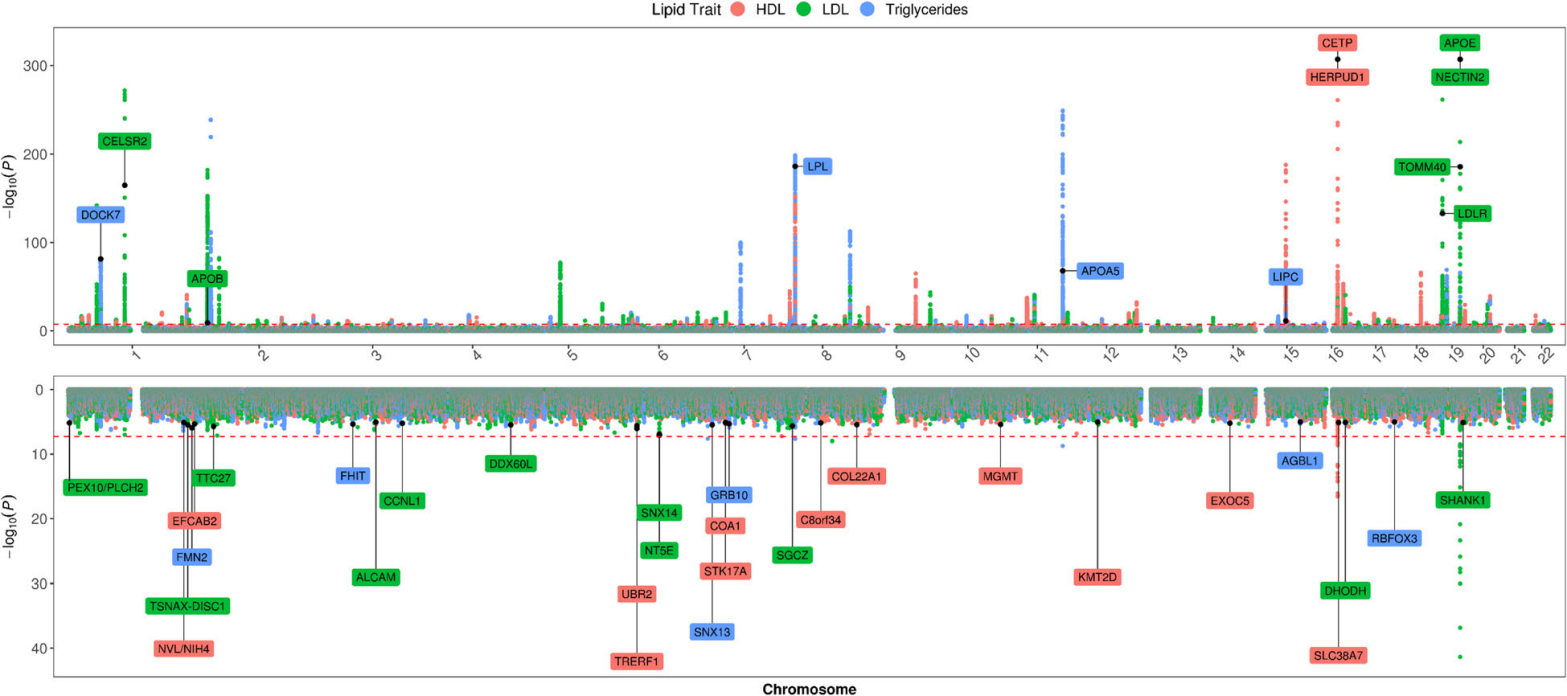
Multi-phenotype, mirrored Manhattan plot of genome-wide association analysis of lipid traits in Willer et al. [31] (top) and the CNICS African American (bottom) cohorts. HDL, high-density lipoproteins, LDL, low-density lipoproteins. In the top panel, gene names are listed for loci with association *p* < E−5 in both cohorts. In the bottom panel, gene names are listed for loci if *p* < 0.01 in the CNICS cohort and *p* > 0.05 in the Willer et al. cohort and there is no overlap between 99% confidence intervals for the corresponding beta coefficients

Figure 1 summarizes GWAS results for HDL, LDL, and triglycerides in PLWH_EA_ alongside previously reported findings in populations of European ancestry [31]. We confirmed strong associations exceeding genome-wide statistical significance of variation in *APOE* (apolipoprotein E), *CETP* (Cholesteryl Ester Transfer Protein) with HDL levels; *APOE* and *APOC1* (apolipoprotein C1) with LDL levels, and *APOA5* (apolipoprotein A5), *BUD13* (BUD13 Homolog), and *TRIB1* (Tribbles Pseudokinase 1) with triglyceride levels in PLWH_EA_ (Fig. 1, top panel; Additional file 2: Table S4). Additional associations at *p* < 1E−5 in both HIV and no-HIV cohorts were detected in other previously reported lipid-related genes, including *LIPC* (Lipase C) and *AQP9* (Aquaporin 9) for HDL; *NECTIN2* (Nectin Cell Adhesion Molecule 2), *CELSR2* (Cadherin EGF LAG Seven-Pass G-Type Receptor 2), *PSRC1* (Proline And Serine Rich Coiled-Coil 1), *APOC4*-*APOC2* (apolipoprotein C4, C2), and *TOMM40* (Translocase Of Outer Mitochondrial Membrane 40) for LDL; and *LPL* (Lipoprotein Lipase), *ZPR1* (Zinc Finger Protein 259), and *SLC18A1* for triglycerides (Fig. 1, top panel; Additional file 2: Table S4). Furthermore, we identified variants that were significant in GWAS_HIV_ but not in GWAS_GEN_, despite having sufficient statistical power (Fig. 1, bottom panel; Additional file 2: Table S5). Specifically, we identified 12 independent loci associated with HDL levels, including intronic variants in *TMTC2*, *CYP2B6*, *GRM7*, *BARX2*, *IGF2BP1*, *CEMIP*, *TNFAIP8*; 11 independent loci associated with LDL levels, including intronic variants in *LBR*, *PRKG1*, *RCOR1*, *TNIP1*, *PRKAG2*, and seven independent loci associated with triglyceride levels, including variants in *SBK1*, *GPR156*, and *CPA6* (Additional file 3: Table S5). In a subgroup analysis of PLWH_AA_, in addition to replicating previously reported associations of *APOE*, *TOMM40*, and *NECTIN2* with LDL, *HERPUD1*/*CETP* with HDL, and *APOA5* with triglycerides at the genome-wide significance level, and of *APOB*, *CELSR2*, and *LDLR* with LDL and *LPL*, *LIPC*, and *DOCK7* with triglyceride levels at *p* < E−5 (Fig. 2, top panel, Additional file 4: Table S6), we found lipid-related loci that were unique to PLWH_AA_ (Fig. 2, bottom panel, Additional file 5: Table S7). Specifically, we identified 18 independent HIV-specific loci associated with HDL, 11 with LDL, and seven with triglyceride levels in PLWH_AA_ at *p* < E−5, including intergenic variants in *CPA6*, previously associated with total cholesterol [57] and T2D [58] in individuals of African ancestry, and *PRKG1* linked to body composition [59]. Lastly, we provide further evidence suggesting sex-specific effects of lipid-related SNPs. While none of these associations achieved genome-wide statistical significance (Additional file 6: Table S8), as a group, the corresponding genes were enriched in the visceral fat deposits and the metabolic syndrome pathways using BioCarta as implemented in Enrichr [49].

### Gene set enrichment analysis

**Fig. 3:**
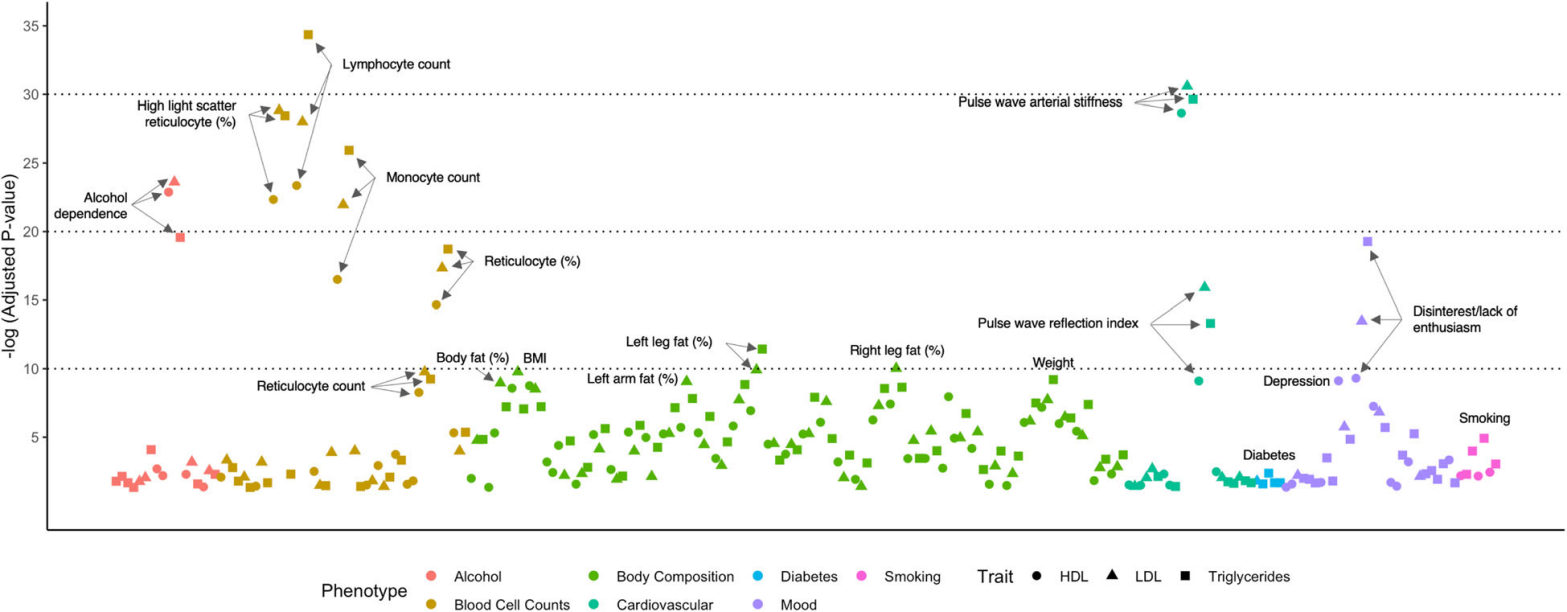
Gene set enrichment analysis of HIV-specific susceptibility loci. Statistical overrepresentation of HIV-specific variants (GWAS_HIV_ *p* < 0.01, GWAS_GEN_ *p* > 0.05, and no overlap between 99% confidence intervals of the corresponding beta coefficients) from GWAS_HIV_ of HDL, LDL, and triglycerides was tested among numerous phenotype terms in the UK Biobank GWAS (version 1) gene set library. The *y*-axis is the negative log_10_ of the adjusted *p* values for each enriched gene set term. The adjusted *p* values were calculated using the Benjamini-Hochberg method for correction for multiple hypotheses testing

Gene set enrichment analysis was performed using genes containing HIV-specific susceptibility loci identified through GWAS_HIV_ of HDL (599 genes), LDL (595 genes), and triglycerides (678 genes). We identified several significantly enriched terms in the UK Biobank GWAS (version 1) gene set library (Fig. 3). Several top enriched terms were associated with blood cell counts, body composition, fat measurements and distribution, hypertension, diabetes, mood changes, and behavioral risk factors, such as alcohol dependence and smoking. Several of these enriched terms were statistically significant in all three gene set enrichment analyses, i.e., using HIV-specific variants from GWAS_HIV_ of HDL, LDL, and triglycerides (Fig. 3).

### Expression quantitative trait loci

Given the association between HIV-specific lipid-related loci and immune cell counts (Fig. 3), we compared the proportion of eSNPs among the HIV-specific SNPs with the proportion of eSNPs among all remaining SNPs in various CD14+ monocyte eQTL data sets (at basal condition, IFN-γ-induced, LPS-induced for 2-h, and LPS-induced for 24-h). The eSNPs were significantly enriched among the HIV-specific SNPs for HDL and LDL (*p* < 0.01) for all conditions except for basal condition for LDL SNPs (Additional file 1: Table S9 and Fig. S1). For triglycerides, the enrichment was significant only in the non-induced cells.

### PRS analysis

**Fig. 4:**
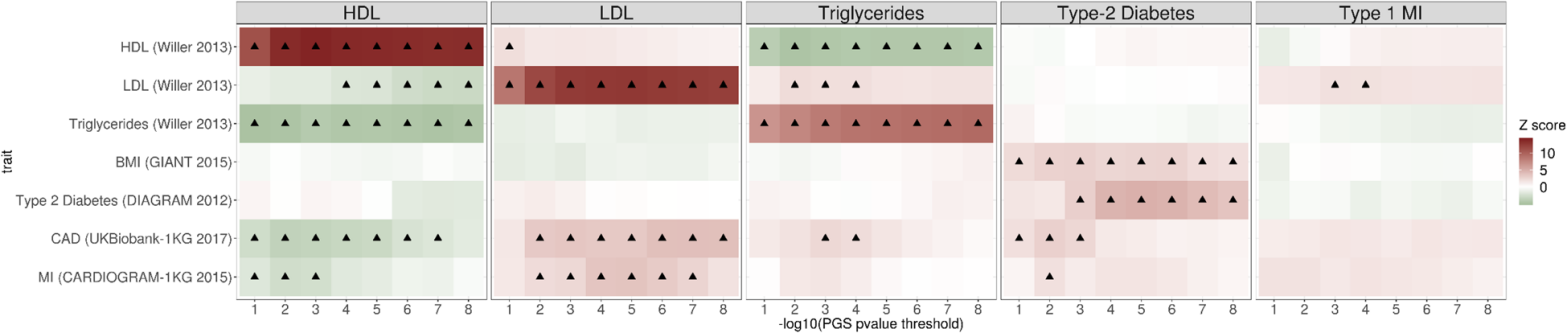
Heat map of polygenic risk scores in the CNICS HIV cohort (European American and African American sub-cohorts combined). The scores were generated using various *p* value cutoffs and SNP-level effect estimates from previously published genome-wide association analyses for each trait/disease phenotype and genotyped and imputed data from the CNICS HIV cohort. The associations marked with “▲” are significant at 10% false discovery rate

We first tested the association of various lipid levels and risk of MI or T2D in CNICS patients with PRS for corresponding traits and diseases derived from GWAS_GEN_ (Additional file 1: Table S2) at eight different GWAS *p* value thresholds. We detected highly significant correlations between PRS for lipid traits (HDL, LDL, and triglycerides) and corresponding phenotypes (e.g., PRS_HDL_ and plasma HDL; Fig. 4). Furthermore, as expected, measured HDL levels were inversely correlated with PRS for LDL, triglycerides, and CAD. Measured LDL levels were positively associated with PRS for CAD and PRS for MI. T2D diagnosis was associated with higher PRS for BMI and CAD. There was a trend toward higher PRS for LDL associated with the risk of MI diagnosis.

**Fig. 5:**
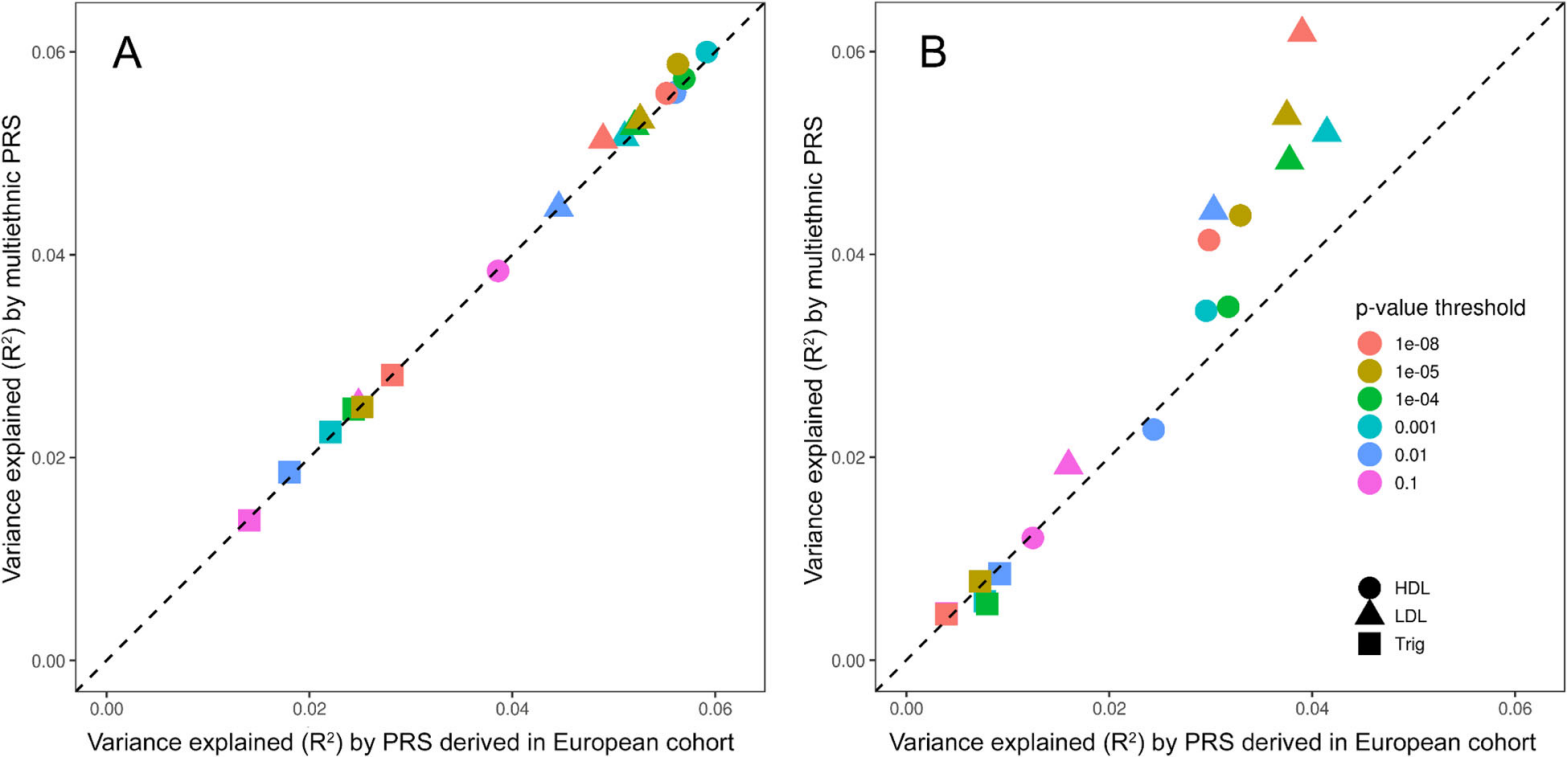
Scatter plot comparing mean variance explained (*R*^2^) by polygenic risk scores (PRS) for lipid traits in African American and European American people living with HIV. *y*-axis: multiethnic PRS derived in HIV cohort. *x*-axis: PRS derived in the general population of European ancestry [31]. **a** European American PLWH. **b** African American PLWH. HDL, high-density lipoproteins; LDL, low-density lipoproteins; Trig, triglycerides

For each lipid trait, we compared the variance explained (adjusted *R*^2^) by the PRS_GEN_ [31] versus multiethnic PRS_HIV_ separately in PLWH_EA_ and PLWH_AA_ (Fig. 5). PRS_GEN_ explained up to 6% of the genetic variance in PLWH_EA_ (Fig. 5a, *x*-axis), but only up to 4% in the PLWH_AA_ sub-cohort (Fig. 5b, *x*-axis). Among the lipid traits, the largest variance explained by PRS_GEN_ was for HDL in PLWH_EA_ and for LDL in PLWH_AA_, whereas the smallest was for triglycerides. Moreover, in PLWH_AA_, using the multiethnic PRS_HIV_ increased the *R*^2^ for LDL across all *p* value thresholds and for HDL, especially when variants with more stringent *p* values were included. In PLWH_EA_, PRS_HIV_ performed as well as PRS_GEN_, with the highest *R*^2^ recorded for HDL across most of *p* value thresholds (Fig. 5).

**Fig. 6:**
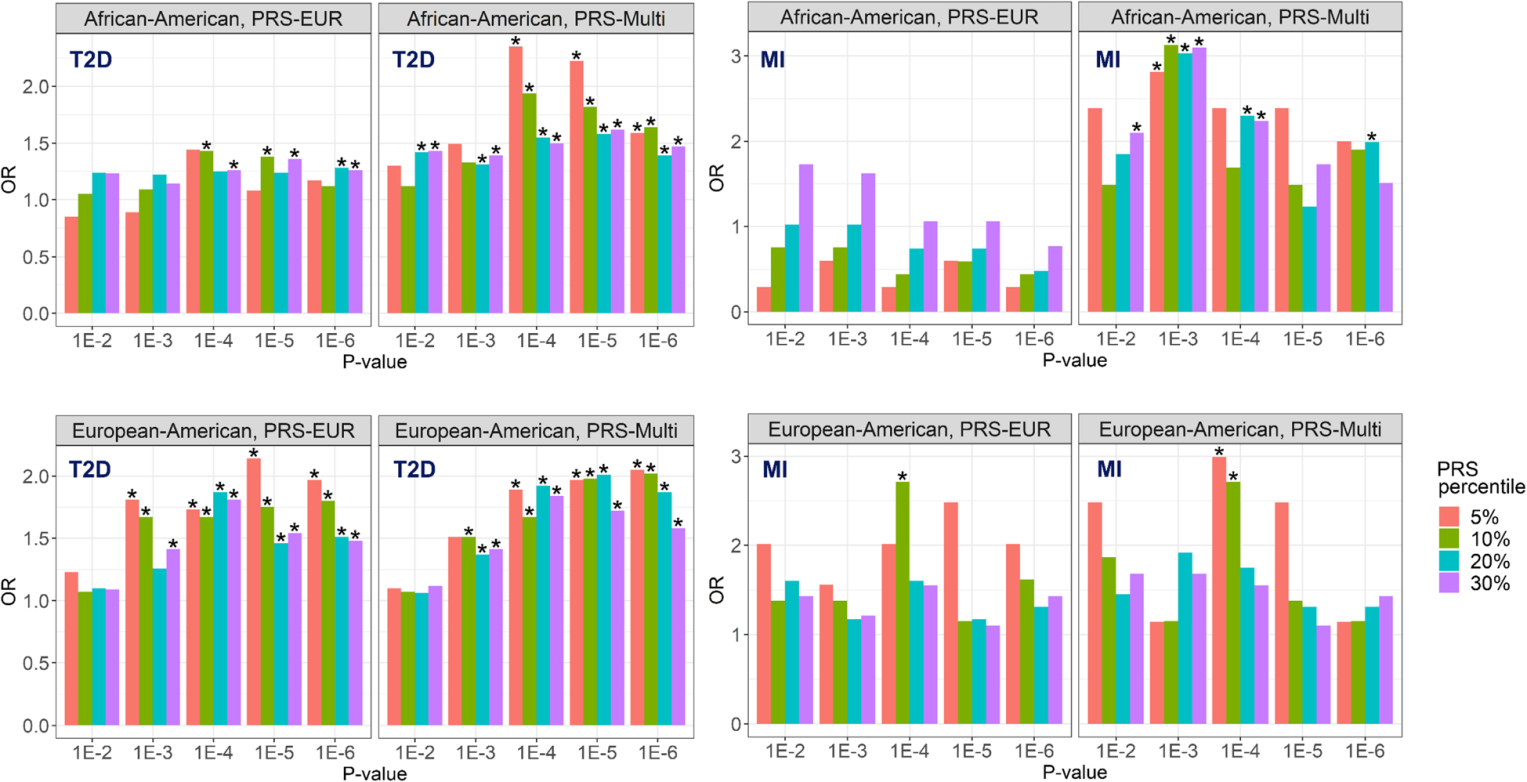
Risk stratification for various polygenic risk score thresholds in European American and African American people living with HIV. OR, odds ratio. PRS-EUR, polygenic risk score derived based on the regression coefficients estimated in a European ancestry population [31]. PRS-Multi, multiethnic PRS. T2D, type 2 diabetes. MI, myocardial infarction. Asterisks denote ORs with false discovery rate-adjusted *p* < 0.05

Lastly, to determine the predictive value of different PRS in the presence of HIV infection, we estimated the risk of T2D and MI among PLWH with the highest PRS_GEN_ (PRS_GEN_ for T2D and PRS_GEN_ for MI, respectively) or the highest multiethnic PRS_HIV_ (PRS_HIV_ for T2D and PRS_HIV_ for MI, respectively). For T2D, PLWH_EA_ at the top 5% of PRS_GEN_T2D_ had an up to 2.14-fold increased risk depending on the GWAS *p* value threshold used for derivation compared to the remaining 95% (Fig. 6, Additional file 7: Table S10). Stratification based on PRS_GEN_ for T2D was unable to distinguish PLWH_AA_ at higher risk of T2D. However, PLWH_AA_ at the top 5% of the multiethnic PRS_HIV_T2D_ had an up to 2.35-fold increased risk (Additional file 7: Table S10). Importantly, although PRS_GEN_ for MI was not predictive of MI risk in PLWH_AA_, patients at the top 5–30% of the multiethnic PRS_HIV_ for MI had a consistently increased risk of MI at various GWAS *p* value thresholds (Additional file 7: Table S10). Neither PRS_GEN_ nor PRS_HIV_ demonstrated any predictive ability for MI risk in PLWH_EA_.

## Discussion

In the largest genetic study in an ethnically diverse cohort of PLWH to date, we confirmed the role of numerous susceptibility loci previously associated with lipid levels in the general population of European descent [31]. In addition, we detected variants uniquely associated with lipid traits in GWAS_HIV_ and not in the large well-powered GWAS_GEN_ of 188,577 individuals [31]. These HIV-specific loci were particularly enriched in eQTLs in basal and induced monocytes and associated with blood cell counts, body metabolism, mood disorders, and predisposition to risky behaviors. Lastly, we demonstrated a predictive value of PRS derived from GWAS_GEN_ in stratifying PLWH_EA_ to distinguish individuals at a higher risk of developing T2D, while top percentiles of multiethnic PRS derived from GWAS_HIV_ and not PRS_GEN_ were associated with increased risk of T2D or MI in PLWH_AA_.

Earlier targeted genotyping studies in general population have reported the role of genome-wide significant susceptibility loci in cardiometabolic traits in PLWH. Specifically, GWAS-validated SNPs in the *APOE*, *APOB*, *LDLR*, and other genes have been demonstrated to contribute to dyslipidemia in the presence of HIV infection [60]. Also, several SNPs and genetic regions common across HIV-positive and HIV-negative women have been detected in association with carotid artery intima-media thickness, a subclinical marker of atherosclerosis [61]. In a series of unbiased GWAS of lipid traits, we confirmed genetic association with previously reported variants in several apolipoprotein-coding genes (*APOE*, *APOC1*, *APOC2*, *APOC4*, and *APOA5*), *CETP*, *LPL*, *BUD13*, *AQP9*, and *CELSR2*, among many others (Fig. 1, Additional file 2: Table S4).

Additionally, we detected numerous loci that were associated with lipid traits in the PLWH_EA_, but showed no significant signal in the large lipid GWAS conducted in a cohort of European ancestry [31] (Fig. 1, Additional file 3: Table S5). A few small GWAS studies performed in HIV-infected cohorts have identified loci associated with carotid atherosclerosis [26], subcutaneous adipose tissue volume [25], and fat loss [24]. In our study, many of the lipid-related susceptibility loci identified in GWAS_HIV_ were also linked by previous studies to HIV viral load [62], susceptibility [63], control [64], smoking behavior [65–67], alcohol dependence [64, 65, 68–70], and cannabis dependence [71–73], more common in PLWH than in individuals without HIV, suggesting the contribution of additional genetic variants associated with HIV infection and adverse lifestyle behaviors to dyslipidemia in this population. Importantly, HIV-specific lipid-related variants were also significantly enriched among the loci associated with blood cell counts, body composition, lifestyle risk factors (alcohol dependence and smoking), and mood disorders (Fig. 3). These findings are consistent with previous reports showing a positive correlation between lymphocyte count and LDL cholesterol levels [74]. Moreover, a shared link has been established between CAD risk and reticulocyte indices, where increased hemolysis associated with high reticulocyte counts may lead to oxidative stress and inflammation [75]. Additionally, a longitudinal relationship of depressive and anxiety symptoms with dyslipidemia and abdominal obesity has been reported [76], which can be partially explained by chronic low-grade inflammation and smoking [77]. While HIV-associated chronic inflammation has long been considered a risk factor of CVD in PLWH [78], our findings suggest that genetic variants may lead to further immune perturbations that contribute to cardiometabolic risk, especially in the presence of HIV infection. Furthermore, when we screened eQTLs in basal and induced CD14+ monocytes of healthy volunteers of European ancestry [52] for the presence of HIV-specific loci, we found significant enrichment for lipid-associated variants, further supporting a functional role of these loci in gene expression regulation of dyslipidemia in the presence of HIV infection. Validation in an independent cohort will be needed to verify the effect of HIV-specific loci on cardiometabolic diseases.

We conducted subgroup analyses to identify lipid-related genetic loci that are unique to PLWH_AA_ (Fig. 2) or act in a sex-specific manner (Additional file 6: Table S8). While none of the associations reached genome-wide significance, we identified a number of genes that have been previously associated with total cholesterol [57] and T2D [58] in individuals of African ancestry, or linked to body composition [59]. The sex-specific genes as a group were enriched in the visceral fat deposit and the metabolic pathways. Additional analyses will be required to dissect the ancestry and sex-specific effects of these variants on metabolic traits in the presence of HIV infection.

Given the polygenic nature of CAD and its numerous risk factors, PRS-based assessment of the genetic burden across multiple susceptibility loci has demonstrated greater predictive value for disease risk and drug response than individual variants [33]. A recent study in a non-HIV cohort has shown that the CAD risk associated with a high polygenic load for lipid-increasing variants was proportional to their impact on lipid levels [79]. We showed a significant correlation of PRS for lipid traits, T2D, and MI generated based on the large European GWAS_GEN_ (Additional file 1: Table S2) with respective phenotypes in PLWH (Fig. 4). Similar to the general population, in PLWH, we observed a positive association of PRS for CAD and PRS for MI with LDL and a negative association with HDL. Our results suggest that lipid PRS could point to modifiable risk factors in the presence of HIV infection, providing additional guidance for clinical application.

However, the variance explained by PRS derived from general (predominantly European) populations in PLWH_EA_ was > 30% lower than that explained in PLWH_AA_ (~ 6% vs. < 4%). This finding is consistent with previous studies showing that PRS calculated using effect estimates from European GWAS were not generalizable to the African ancestry population [80]. Therefore, we calculated a multiethnic PRS, shown to significantly improve disease prediction accuracy in a non-European cohort [56], by applying weights in both EA and AA GWAS in CNICS using ten-fold cross-validation. Multiethnic PRS_HIV_ outperformed PRS_GEN_ in PLWH_AA_, especially for HDL, but not in PLWH_EA_ (Fig. 5).

Of note, stratification based on PRS_GEN_ for T2D was able to distinguish PLWH that were at a higher risk of T2D, with EA at the top 5% having a more than two-fold increased risk; the impact of PRS_GEN_ for T2D on T2D risk in AA was less obvious (Fig. 6; Additional file 7: Table S10). A 2.75-fold increased risk of T2D in individuals of European ancestry at the top 5% of PRS for T2D has been previously reported [21]. However, the multiethnic PRS for T2D significantly improved T2D risk stratification in AA, but not in EA PLWH (Fig. 6).

In addition, while PRS_GEN_ for MI was unable to significantly stratify MI risk in either ethnic subgroup, multiethnic PRS_HIV_ demonstrated over a 3-fold increased risk in PLWH_AA_. Multiethnic PRS_HIV_ for MI largely unchanged the disease risk prediction in PLWH_EA_. In a much larger European ancestry non-HIV cohort, a 1 standard deviation higher PRS is associated with a 33% increased risk of incident MI in participants without CAD [81]. Taken together, our findings suggest that, while the large GWAS in ethnically and racially diverse cohorts should substantially contribute to the accuracy of PRS prediction in PLWH, in the absence of such studies, multiethnic scores are feasible alternatives to identify at-risk individuals. Given that medications and intensive lifestyle interventions prevent or postpone the progression to T2D and MI [82, 83], ascertainment of PLWH with high PRS may provide an opportunity to target these interventions with increased precision.

This study has some limitations. In the general population-based cohorts used in our analyses, HIV infection-related information may not have been collected or considered during recruitment or analysis. Therefore, it is possible to have an unknown number of PLWH in these cohorts. However, the rate of HIV infection in the US population is relatively low (~ 1 in 300), and inclusion of such individuals in our analyses would bias the results toward the null. We controlled for ART presence or absence and made no distinctions across ART regimens. A thorough investigation of the effects of ART on lipids, which is a rapidly evolving field, is a big task and beyond the scope of the present analysis. Future investigations may be able to refine some of the work done in our study. We performed analyses of PRS for BMI but did not analyze the observed BMI. Many factors are associated with BMI among PLWH, including body morphology disorders and lifestyle, and fully analyzing these characteristics was beyond the scope of this study. Future work should elucidate relationships with the observed BMI. Additionally, we used the same cohort for multiethnic PRS derivation and validation; however, we do not expect over-fitting to be a concern given the small number of mixing weights optimized (up to 2) relative to the target sample size (> 3000) and given our use of adjusted *R*^2^ as the evaluation metric, similar to previously reported analyses [56]. In order to minimize the possibility of an inflated *R*^2^ prediction due to shared population stratification or familial/distant relatedness [84], we used ancestry-adjusted regression coefficients for PRS computation and ten-fold cross-validation. Despite being the largest genetic study reported in PLWH, the number of MI cases was too small to provide sufficient statistical power to assess the clinical impact of PRS. Nevertheless, we were able to demonstrate that the use of multiethnic PRS in PLWH outperformed PRS derived in largely European populations, especially for PLWH_AA_. Going forward, meta-analyses of PLWH cohorts should allow for validation of our findings and help assess the clinical impact of the genetic burden on disease risk.

## Conclusions

In summary, we demonstrated that in addition to genetic loci in the lipid metabolism genes previously linked to dyslipidemia and other CAD-related risks in the general population, there are other genetic factors that can impact lipid levels by further enhancing inflammation and predisposing to mood disorders and risky behaviors, thereby contributing to dyslipidemia in the presence of HIV infection. Comprehensive polygenic risk profiling identified PLWH to be at a several-fold increased risk of T2D or MI, which may help increase the precision of ascertaining those at high risk for targeted interventions.

## Supplementary information

**Additional file 1:** Table S1. A summary of genotyping and imputation platforms utilized to generate the final study data set. Table S2. Description of previously published genome-wide association studies (GWAS) in European populations that were used in polygenic risk score analyses. Table S3. Number of variants used to derive various polygenic risk scores for different traits and diseases. Table S9. Number of expression quantitative trait loci controlled by HIV-specific loci in CD14+ monocytes from Fairfax et al. [51]. Fig. S1. Fraction of expression quantitative trait loci among the HIV-specific loci (see the Methods section for details) compared to all other loci. HDL, high-density lipoprotein; LDL, low-density lipoprotein; TG, triglycerides; SNP, single nucleotide polymorphism. CD14, CD14+ monocytes at baseline; INF, activated CD14+ monocytes following induction with interferon-γ; LPS2, activated CD14+ monocytes following a 2-h induction with lipopolysaccharide; LPS24, activated CD14+ monocytes following a 24-h induction with lipopolysaccharide. SNP, single nucleotide polymorphism.

**Additional file 2: **Table S4. List of variants significantly associated with lipid levels in European Americans in the CNICS HIV cohort (p < E-5) and in Willer et al. [31] (*p* < 0.05).

**Additional file 3: **Table S5. List of variants significantly associated with lipid levels in European Americans in the CNICS HIV cohort (p < E-5), non-significant in Willer et al. [31] (*p* > 0.05) and with no overlap between 99% confidence intervals for beta coefficients between CNICS and Willer et al.

**Additional file 4:** Table S6. List of variants significantly associated with lipid levels in African Americans in the CNICS HIV cohort (p < E-5) and in Willer et al. [31] (p < 0.05).

**Additional file 5:** Table S7. List of variants significantly associated with lipid levels in African Americans in the CNICS HIV cohort (p < E-5), non-significant in Willer et al. [31] (p > 0.05) and with no overlap between 99% confidence intervals for the beta coefficients between CNICS and Willer et al.

**Additional file 6:** Table S8. List of variants significantly associated with lipid levels in European American females (p < E-5) but not in males (p > 0.05) in the CNICS cohort with no overlap between 99% confidence intervals for beta coefficients between females and males.

**Additional file 7:** Table S10. Prevalence and clinical impact of high European-based and multiethnic polygenic risk scores for type-2 diabetes (T2D) and myocardial infarction (MI) in people living with HIV.

## Data Availability

The datasets generated and analyzed during the current study are available from the authors on reasonable request.

## Abbreviations

AA: African American
AIDS: Acquired immune deficiency syndrome
ART: Antiretroviral therapy
BMI: Body mass index
CAD: Coronary artery disease
CNICS: Centers for AIDS Research Network of Integrated Clinical Systems
CVD: Cardiovascular disease
EA: European American
eQTL: Expression quantitative trait locus
FDR: False discovery rate
GWAS: Genome-wide association study
HDL: High-density lipoprotein cholesterol
HIV: Human immunodeficiency virus
IRB: Institutional Review Board
KEGG: Kyoto Encyclopedia of Genes and Genomes
LD: Linkage disequilibrium
LDL: Low-density lipoprotein cholesterol
MEGA: Multiethnic Global Array
MI: Myocardial infarction
PCA: Principal components analysis
PLWH: People living with HIV
PLWH_AA_: African American sub-cohort of people living with HIV
PLWH_EA_: European American sub-cohort of people living with HIV
PRS: Polygenic risk score
QC: Quality control
SNP: Single nucleotide polymorphism
T2D: Type 2 diabetes
