# Correction to: Genetic architecture of cardiometabolic risks in people living with HIV

**DOI:** 10.1186/s12916-021-01976-9

**Published:** 2021-05-05

**Authors:** Haoxiang Cheng, Anshuman Sewda, Carla Marquez-Luna, Sierra R. White, Bridget M. Whitney, Jessica Williams-Nguyen, Robin M. Nance, Won Jun Lee, Mari M. Kitahata, Michael S. Saag, Amanda Willig, Joseph J. Eron, W. Christopher Mathews, Peter W. Hunt, Richard D. Moore, Allison Webel, Kenneth H. Mayer, Joseph A. Delaney, Paul K. Crane, Heidi M. Crane, Ke Hao, Inga Peter

**Affiliations:** 1 Department of Genetics and Genomic Sciences, Icahn School of Medicine at Mount Sinai, 1425 Madison Avenue, New York, NY 10029 United States of America; 2 Institute of Health Management Research, IIHMR University, Jaipur, Rajasthan India; 3 The Charles Bronfman Institute for Personalized Medicine, Icahn School of Medicine at Mount Sinai, New York, NY United States of America; 4 Department of Epidemiology, University of Washington School of Public Health, Seattle, WA United States of America; 5 Department of Medicine, University of Washington School of Medicine, Seattle, WA United States of America; 6 Center for AIDS Research, University of Washington, Seattle, WA United States of America; 7 School of Medicine, University of Alabama at Birmingham, Birmingham, AL United States of America; 8 Department of Medicine, School of Medicine, University of North Carolina at Chapel Hill, Chapel Hill, NC 27514 United States of America; 9 Department of Medicine, University of California San Diego, San Diego, CA United States of America; 10 Division of Experimental Medicine, University of California San Francisco, San Francisco, CA United States of America; 11 Department of Medicine, Johns Hopkins University, Baltimore, MD United States of America; 12 Department of Epidemiology, |Johns Hopkins University, Baltimore, MD United States of America; 13 Frances Payne Bolton School of Nursing, Case Western Reserve University, Cleveland, OH United States of America; 14 The Fenway Institute at Fenway Health, Boston, MA United States of America

**Correction to: BMC Med 18, 288 (2020)**

**https://doi.org/10.1186/s12916-020-01762-z**

The original article [1] contained an error whereby first author, Haoxiang Cheng’s name was displayed incorrectly. This has since been corrected.
