# Supplementary material for: Genetic architecture of cardiometabolic risks in people living with HIV
Source: BMC Med. 2020 Oct 28;18:288. doi: 10.1186/s12916-020-01762-z (PMC7592520; doi:10.1186/s12916-020-01762-z)
Supplement: Supplementary file 1 — Additional file 1: Table S1. A summary of genotyping and imputation platforms utilized to generate the final study data set. Table S2. Description of previously published genome-wide association studies (GWAS) in European populations that were used in polygenic risk score analyses. Table S3. Number of variants used to derive various polygenic risk scores for different traits and diseases. Table S9. Number of expression quantitative trait loci controlled by HIV-specific loci in CD14+ monocytes from Fairfax et al. [51]. Fig. S1. Fraction of expression quantitative trait loci among the HIV-specific loci (see the Methods section for details) compared to all other loci. HDL, high-density lipoprotein; LDL, low-density lipoprotein; TG, triglycerides; SNP, single nucleotide polymorphism. CD14, CD14+ monocytes at baseline; INF, activated CD14+ monocytes following induction with interferon-γ; LPS2, activated CD14+ monocytes following a 2-h induction with lipopolysaccharide; LPS24, activated CD14+ monocytes following a 24-h induction with lipopolysaccharide. SNP, single nucleotide polymorphism. [file 12916_2020_1762_MOESM1_ESM.docx]

**Additional file 1: SUPPORTING INFORMATION**

**Genetic architecture of cardiometabolic risks in people living with HIV**

Haoxiang Chang, Anshuman Sewda, Carla Marquez-Luna*,* Sierra R. White, Bridget M. Whitney, Jessica Williams-Nguyen, Robin M. Nance, Won Jun Lee, Mari M. Kitahata, Michael S. Saag, Amanda Willig, Joseph J. Eron, W Christopher Mathews, Peter W. Hunt, Richard D. Moore, Allison Webel, Kenneth H. Mayer, Joseph A. Delaney, Paul K. Crane, Heidi M. Crane, Ke Hao, and Inga Peter

**Table S1.** A summary of genotyping and imputation platforms utilized to generate the final study data set.

| **Illumina Genotyping Platform** | **Number of Assayed SNPs** | | **Number of Imputed SNPs** | |
| --- | --- | --- | --- | --- |
|  | SNPs called | After QC^a^ | SNPs Imputed^b^ | After QC^c^ |
| MEGA | 1,705,969 | 1,523,448 |  |  |
| MEGA^EX^ | 2,036,060 | 1,788,482 | 291,989,249 | 13,620,258 |
| MEG | 1,779,819 | 1,752,471 |  |  |

^a^Single nucleotide polymorphism (SNP) quality control (QC) criteria: SNP call rate ≥ 90% and Hardy-Weinberg p-value ≥ 1E-20, where Hardy-Weinberg p-values were computed separately in European Americans and African Americans. ^b^Imputation was performed using Trans-Omics for Precision Medicine (TOPMed) data as the reference. ^c^SNP filtering criteria: Liftover to human genome build hg19 or Genome Reference Consortium Human Build 37 (GRCh37) was performed. SNPs with imputation r2 ≥ 0.3 and minor allele frequency (MAF) ≥ 1% were kept in the data, where MAF was computed separately in individuals of European and African ancestry. MEG, Multi-Ethnic Global Array.

**Table S2.** Description of previously published genome-wide association studies (GWAS) in European populations that were used in polygenic risk score analyses.

| European GWAS Consortium^a^ | Sample size^b^ | Number of  single nucleotide polymorphisms^c^ | Polygenic Risk Score  Analysis Trait^d^ |
| --- | --- | --- | --- |
| GLGC | 188,577 | 2,447,441 | HDL, LDL, and triglycerides |
| GIANT | 322,154 | 2,554,623 | Body mass index |
| DIAGRAM | Cases: 12,171  Controls: 56,862 | 2,473,441 | Type 2 diabetes |
| CARDIoGRAMplusC4D | Cases: 60,801  Controls: 123,504 | 9,455,441 | Myocardial infarction |
| UK Biobank | Cases: 71,602  Controls: 260,875 | ~9,100,000 |  |

^a^Previously published European GWAS sources for polygenic risk score analysis: GLGC, Global Lipids Genetics Consortium; GIANT consortium, Genetic Investigation of ANthropometric Traits consortium; DIAGRAM consortium, DIAbetes Genetics Replication And Meta-analysis consortium; CARDIoGRAMplusC4D, Coronary ARtery DIsease Genome wide Replication and Meta-analysis plus the Coronary Artery Disease Genetics consortium; and UKBiobank CardioMetabolic Consortium. ^b^The number of European ancestry individuals in each consortium, the controls in CARDIoGRAMplusC4D were primarily of European ancestry. ^c^The number of imputed single nucleotide polymorphisms in each consortium. ^d^Trait for which the GWAS summary statistics from each consortium were used to derive polygenic risk scores.

**Table S3.** Number of variants used to derive various polygenic risk scores for different traits and diseases

| **Polygenic Risk Score** | **GWAS**  **P-value threshold** | **HDL** | **LDL** | **TG** | **T2D** | **MI** |
| --- | --- | --- | --- | --- | --- | --- |
| **Derived in individuals of European ancestry** | 1E-1 | 47,416 | 47,509 | 49,003 | 59,914 | 75,538 |
|  | 1E-2 | 6,972 | 6,723 | 7,089 | 8,894 | 10,127 |
|  | 1E-3 | 2,108 | 1,969 | 1,982 | 1,671 | 1,722 |
|  | 1E-4 | 1,104 | 1,035 | 1,011 | 459 | 429 |
|  | 1E-5 | 754 | 721 | 695 | 207 | 182 |
|  | 1E-6 | 583 | 535 | 512 | 124 | 103 |
|  | 1E-7 | 474 | 447 | 394 | 89 | 58 |
|  | 1E-8 | 396 | 376 | 324 | 64 | 37 |
| **Multi-ethnic** | 1E-1 | 194,798 | 196,193 | 196,073 | 208,164 | 213,860 |
|  | 1E-2 | 25,897 | 26,603 | 26,509 | 27,615 | 26,330 |
|  | 1E-3 | 4,416 | 4,605 | 4,426 | 3,860 | 3,801 |
|  | 1E-4 | 1,395 | 1,381 | 1,269 | 696 | 780 |
|  | 1E-5 | 800 | 783 | 728 | 245 | 219 |
|  | 1E-6 | 594 | 552 | 517 | 136 | 108 |
|  | 1E-7 | 482 | 455 | 397 | - | - |
|  | 1E-8 | 402 | 382 | 325 | - | - |

GWAS, genome wide association study. HDL, high-density lipoprotein; LDL, low-density lipoprotein, TG, triglycerides; T2D, type-2 diabetes; MI, myocardial infarction.

**Additional file 2: Table S4.** List of variants significantly associated with lipid levels in European Americans in the CNICS HIV cohort (p < E-5) and in Willer et al. [31] (p < 0.05).

**Additional file 3: Table S5.** List of variants significantly associated with triglyceride levels in European Americans in the CNICS HIV cohort (p < E-5), non-significant in Willer et al. [31] (p > 0.05) and with no overlap between 99% confidence intervals for beta coefficients between CNICS and Willer et al.

**Additional file 4: Table S6.** List of variants significantly associated with triglyceride levels in African Americans in the CNICS HIV cohort (p < E-5) and in Willer et al. [31] (p < 0.05).

**Additional file 5: Table S7.** List of variants significantly associated with lipid levels in African Americans in the CNICS HIV cohort (p < E-5), non-significant in Willer et al. [31] (p > 0.05) and with no overlap between 99% confidence intervals for beta coefficients between CNICS and Willer et al.

**Additional file 6: Table S8.** List of variants significantly associated with lipid levels in European American females (p < E-5) but not in males (p > 0.05) in the CNICS cohort with no overlap between 99% confidence intervals for beta coefficients between females and males.

**Table S9.** Number of expression quantitative trait loci controlled by HIV-specific loci in CD14+ monocytes from Fairfax et al. [51].

| **Trait** | **CD14+ naïve monocytes** | **IFN-induced monocytes** | **LPS 2hr-induced Monocytes** | **LPS 24hr-induced monocytes** |
| --- | --- | --- | --- | --- |
| **LDL** | 76 | 74 | 44 | 67 |
| **TG** | 91 | 96 | 54 | 67 |
| **HDL** | 82 | 69 | 36 | 63 |

HDL, high-density lipoprotein; LDL, low-density lipoprotein; TG, triglyceride; SNP, single nucleotide polymorphism. CD14, CD14+ monocytes at baseline; INF, activated CD14+ monocytes following induction with interferon-γ; LPS2, activated CD14+ monocytes following a 2-hour induction with lipopolysaccharide; LPS24, activated CD14+ monocytes following a 24-hour induction with lipopolysaccharide.

**Additional file 7: Table S10.**  Prevalence and clinical impact of high European-based and multiethnic polygenic risk scores for type-2 diabetes (T2D) and myocardial infarction (MI) in people living with HIV.


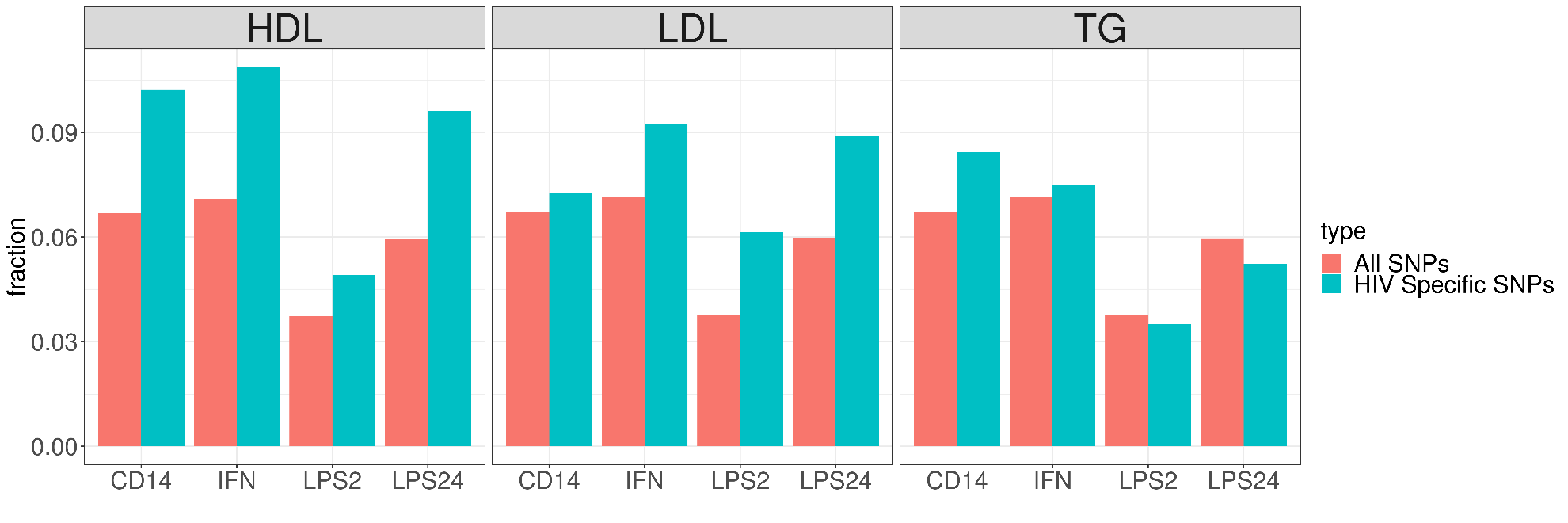


**Fig. S1.** Fraction of expression quantitative trait loci among the HIV-specific loci (see the Methods section for details) compared to all other loci. HDL, high-density lipoprotein; LDL, low-density lipoprotein; TG, triglycerides; SNP, single nucleotide polymorphism. CD14, CD14+ monocytes at baseline; INF, activated CD14+ monocytes following induction with interferon-γ; LPS2, activated CD14+ monocytes following a 2-hour induction with lipopolysaccharide; LPS24, activated CD14+ monocytes following a 24-hour induction with lipopolysaccharide. SNP, single nucleotide polymorphism.
